# Motor abnormalities as a putative endophenotype for Autism Spectrum Disorders

**DOI:** 10.3389/fnint.2013.00043

**Published:** 2013-06-11

**Authors:** Gianluca Esposito, Sergiu P. Paşca

**Affiliations:** ^1^RIKEN Brain Science Institute, Unit for Affiliative Social BehaviorWako-Shi, Saitama, Japan; ^2^Department of Neurobiology, Stanford University School of MedicineStanford, CA, USA

**Keywords:** Autism Spectrum Disorders, motor abnormalities, endophenotype, early assessment, early screening

## Abstract

Autism Spectrum Disorders (ASDs) represent a complex group of behaviorally defined conditions with core deficits in social communication and the presence of repetitive and restrictive behaviors. To date, neuropathological studies have failed to identify pathognomonic cellular features for ASDs and there remains a fundamental disconnection between the complex clinical aspects of ASDs and the underlying neurobiology. Although not listed among the core diagnostic domains of impairment in ASDs, motor abnormalities have been consistently reported across the spectrum. In this perspective article, we summarize the evidence that supports the use of motor abnormalities as a putative endophenotype for ASDs. We argue that because these motor abnormalities do not directly depend on social or linguistic development, they may serve as an early disease indicator. Furthermore, we propose that stratifying patients based on motor development could be useful not only as an outcome predictor and in identifying more specific treatments for different ASDs categories, but also in exposing neurobiological mechanisms.

Autism Spectrum Disorders (ASDs) comprise a complex group of behaviorally defined conditions with core deficits in social communication and the presence of repetitive and restrictive behaviors (American Psychiatric Association, 2000). ASDs are highly comorbid and notably heterogeneous in their clinical presentation. Multiple etiologies have been suggested, but no single genetic or environmental factor can account for more than a small fraction of all cases (Abrahams and Geschwind, 2008). Despite sustained efforts to identify the cell types and circuits that are impaired in ASDs, there remains a fundamental disconnection between the complex clinical features of ASDs and the underlying neurobiological mechanisms. Postmortem brain studies have failed to identify pathognomonic cellular features for ASDs (Pickett and London, 2005; Amaral et al., 2008). Despite reports of high heritability (Abrahams and Geschwind, 2008, 2010), large effect genetic events (copy number variants or *de novo* mutations) are rare, while common genetic variants can explain only a minute fraction of the phenotypic variability (Stein et al., 2013). In addition, environmental contributions have only rarely proved conclusive [e.g., rubella, thalidomide or valproic acid exposure in early pregnancy (Landrigan, 2010)]. While rodent models of ASDs have begun to provide pathophysiological and therapeutic clues, these models have been restricted to rare syndromic or Mendelian forms of ASDs, and have yet to address issues of specificity (i.e., the overlap between genes in ASDs, developmental delay, and schizophrenia) and cross-species clinical validity (Qiu et al., 2012). More recently, cellular reprogramming techniques have emerged as new tools for identifying neuronal phenotypes in cells derived *in vitro* from patients (Marchetto et al., 2010; Paşca et al., 2011; Novarino et al., 2012). However, these cellular investigations will have to be expanded considerably in order to identify common and divergent neuronal phenotypes in idiopathic ASDs cases.

These novel models, as well as the continued accumulation of clinical and genetic data in recent years, underscore a need to develop more reliable means of stratifying ASDs. The identification of discrete, genetically determined disease components, or endophenotypes (Gottesman and Gould, 2003), could prove essential in delineating biologically and therapeutically meaningful classes, adding power to genetic studies and guiding neurobiological investigations. One promising avenue in this direction is a more exhaustive and systematic investigation of motor abnormalities in ASDs.

Motor abnormalities in ASDs span a wide range of dysfunctions, including defects in gross and fine motor control, complex motor sequences (including dyspraxia and deficits in imitation), eye movement abnormalities and motor learning deficits. Pinpointed by Kanner (1943) and Asperger (1944) in their initial case series, these abnormalities were referred as “clumsiness in gait and motor performances” (Kanner and Lesser, 1958). Two decades later, Damasio and Maurer (1978) hypothesized mesolimbic dysfunction as a potential explanation for dyskinetic and dystonic movements observed in patients with ASDs, while others have either described a parkinsonian gait (Vilensky et al., 1981), an ataxic-cerebellar gait (Hallett et al., 1993; see also Nayate et al., 2012) or simply recognized asymmetric patterns of movement and infantile reflexes “gone astray” (Teitelbaum et al., 1998, 2004; Esposito et al., 2009, 2011, 2012). Although a recent meta-analysis confirmed the presence of substantial motor coordination deficits in ASDs with a considerable effect size of 1.20 (Fournier et al., 2010), none of the studies to date have identified a single motor symptom as a universal sign or prodrome for ASDs (Yirmiya and Charman, 2010).

Though there may not be a single universal motor sign, several levels of evidence point toward the utility of motor assessments in ASDs, indicating that motor dysfunction may play a central role in elucidating pathophysiological mechanisms and facilitating diagnosis and treatment. We describe them here with an emphasis on highlighting specific commonalties and disparities in the presentation of motor abnormalities that could allow for ASDs stratification.

First, motor abnormalities in ASDs are present early, within the first year of life, and may precede social-communication deficits (Leary and Hill, 1996). For example, Flanagan et al. (2012), reported that head lag during pull-to-sit at the age of 6 months was associated with ASDs at 36 months and was more frequently observed in infants at high-risk for ASDs. Recently, two excellent prospective studies followed early motor symptoms in high-risk subjects. In the first longitudinal study, Landa et al. (2013) assessed 235 children with or without a sibling with ASDs to identify differential trajectories for normative versus early-onset or late-onset ASDs. Interestingly, although development was grossly intact by 6 months, fine motor delay was present as early as 14 months in the late-onset group, and only by 36 months in the early-onset ASDs group. In the second prospective study, Landa et al. (2012) followed ASDs siblings from 6 to 36 months and identified four main trajectory phenotypes: normal development, accelerated development, widespread skill acquisition delay, and receptive language and motor delay. Importantly, in the latter group, receptive language delay resolved by 24 months, while motor abnormalities persisted at 36 months. Taken together, these studies demonstrate that motor development is vulnerable to early delay in patients with ASDs and their siblings and could potentially inform subtype identification.

Second, motor symptoms in ASDs are persistent. Both fine and gross motor impairments are long-term deficits, whose severity is correlated with the degree of social impairment (Freitag et al., 2007). A recent large sample study (Lloyd et al., 2013) showed that in very young children with ASDs, these delays become more pronounced with age, even when controlling for non-verbal problem-solving skills. Additional reports have suggested that gross motor and fine motor symptoms may diminish over the course of life, but even in these cohorts oculomotor impairment and dyspraxia appear to persist (Freitag et al., 2007). These observations suggest that the persistence of motor symptoms could also assist with differential diagnosis. For instance, skill progression in Down syndrome is delayed, but the acquisition of developmental milestones occurs in an orderly manner and these deficits can significantly improve with therapeutic facilitation (Sacks and Buckley, 2003).

Third, there is preliminary evidence indicating that motor abnormalities in ASDs are heritable. For instance, motor delays are common among ASDs siblings and are predictive of communication delays in these individuals (Bhat et al., 2012), making them part of the broader autism phenotype. In addition, bivariate twin analyses indicate that physical clumsiness and autistic-like traits are highly correlated, an association that is most plausibly explained by genetic etiological overlap (Moruzzi et al., 2011). Moreover, a considerable proportion of the genetic variance in ASDs is shared with developmental coordination disorder, a childhood condition characterized by poor motor coordination and clumsiness (Lichtenstein et al., 2010). While not all studies have been able to detect motor skills impairments in unaffected siblings of children with ASDs (Hilton et al., 2012), future prospective studies should dissect more systematically and in larger cohorts the relative genetic contribution to motor abnormalities in ASDs.

Fourth, and perhaps the feature that best makes the case for the assessment of motor abnormalities for ASDs stratification, is the fact that motor development is relatively more quantitative in nature than communicative abilities or social traits. Multiple standardized test batteries that measure motor skills are currently available. For instance, the Mullen Scales of Early Learning evaluates gross motor development from 0 to 33 months, and the Griffiths Mental Development Scales quantify locomotor activity, including the ability to balance and to coordinate and control movements. Multiple studies have shown that these evaluations are reliable and easy to implement. Moreover, they have the potential for becoming screening tools especially if facilitated by video analyses (using computer vision tools as illustrated by Hashemi et al., 2012, for example). Coupled with the early onset of motor abnormalities, described above, the availability of reliable quantitative tools point toward the use of motor development as a more standard metric for patient stratification.

Fifth, is the suggestion that both motor and social-communicative deficits originate from a common etiology and that motor abnormalities would constitute an early window into the pathophysiology of ASDs. Although this assertion has not been tested systematically, we know a significant amount about the physiology of the motor system, and it is conceivable that neurobiological insights will be gained from investigating motor development in ASDs. Clinical and physiological studies indicate multiple levels of biological impairment in ASDs, from the vestibular brainstem nuclei to the cerebellum, basal ganglia and sensorimotor cortices. Therefore, involvement of individual structures, which are associated with specific subtypes of motor abnormalities, could be used as a stratification criterion. Postmortem brain findings have paved the way by reporting, in some ASDs patients, Purkinje cell deficits in the cerebellum (Bauman and Kemper, 1985; Arin et al., 1991; Bailey et al., 1998; Whitney et al., 2008; Fatemi et al., 2012) and hypoplasia of its vermal lobules VI–VII (Courchesne et al., 1994), an enlarged caudate nucleus (Langen et al., 2007) and a delayed functional specialization of the motor cortex (Nebel et al., 2012). One example of how motor abnormalities may inform a mechanistic understanding is the hypothesis of early damage to mirror neuron systems in ASD. According to this model, impairments in ASDs are rooted in the incapacity to assemble and directly grasp the intrinsic goal-directed organization of motor behavior (Cossu et al., 2012).

Sixth, motor abnormalities affect quality of life and correction is likely to improve functioning. Abnormal motor control can have pervasive consequences on the development of multiple skills. Delayed motor development constrains social interactions and impaired social interactions can further constrain motor skill development. Importantly, gross and fine motor skills can be learned and practiced, and although not tested prospectively, motor corrections may improve social-communicative functioning in ASDs (Baranek, 1999).

Lastly, recent studies indicate that motor abnormalities in ASDs may have predictive value. For instance, approximately 70% of high-risk infants (i.e., siblings of ASDs patients) who presented with early motor delays later developed deficits in communication (Bhat et al., 2012), while better motor outcome in 2-year-old children with ASDs correlates with better outcomes at 4 years (Sutera et al., 2007).

Taken together, these multiple lines of evidence underscore the need for more systematically assessing motor development in ASDs patients. With few exceptions (Provost et al., 2007; Ozonoff et al., 2008), most studies investigating motor development in ASDs report abnormalities at some levels (vestibular, fronto-striatal, cerebellar, cortical) and of a certain severity. Importantly, the standard deviations for the measured variables in these studies are always larger in the ASDs group, highlighting that at an individual level some children with ASDs are definitely atypical, while others are probably not remarkably different than their matching controls (Vernazza-Martin et al., 2005; Rinehart et al., 2006a,b,c; Esposito and Venuti, 2008). Depending on the task and the cohort, the proportion of ASDs children displaying motor development abnormalities varies. For instance Esposito et al. (2011), found that persistent postural asymmetries were present only in ~40% of children with ASDs. The variability in these deficits across the spectrum is a challenge that likely reflects the clinical and etiological heterogeneity of ASDs. At the same time, it constitutes a unique opportunity to identify disease subtypes.

Additional work is clearly needed to conclusively determine how motor abnormalities can contribute to understanding ASDs, while the limitations of existing studies also have to be addressed. For instance, cohorts that up until now have been restricted to high functioning patients should be expanded to reflect the full autism spectrum using objective disease measures (the Autism Diagnostic Observation Schedule—ADOS, Autism Diagnostic Interview, Revised—ADI-R). More prospective studies need to be developed, while retrospective studies should use well-matched controls and siblings. It is also important to explore motor disturbances in novel or cognitively demanding environments and combine these studies with genetic analyses and neuroimaging. Peculiar phenomena associated with ASDs, such as *kinesia paradoxa* during which motor function can appear smooth and seamless during fixation on one task, deserve more attention (Leary and Hill, 1996; Rinehart et al., 2006a,b,c). In addition, the confounding role of medications (antipsychotics, antidepressants, stimulants, mood stabilizers), which are so commonly prescribed in these patients, should be rigorously investigated in the context of behavioral and motor abnormalities. Benefits could also come from the development and implementation of novel, easy to use, standardized scales that could streamline the collection of motor developmental data and allow for large-scale analyses.

In conclusion, motor abnormalities in ASDs are early and persistent clinical signs, which, due to their heritability, can serve as disease endophenotypes. In addition, these abnormalities can be reliably quantified and, if improved, are likely to benefit the overall functioning of the patient. When viewed as an endophenotype, motor abnormalities have the potential to stratify ASDs into more tractable conditions leading to more productive etiological explorations, better clinical trials, and perhaps earlier detection and outcome prediction.

## Conflict of interest statement

The authors declare that the research was conducted in the absence of any commercial or financial relationships that could be construed as a potential conflict of interest.
